# The mediator role of communication skill in the relationship between empathy, team cohesion, and competition performance in curlers

**DOI:** 10.3389/fpsyg.2023.1115402

**Published:** 2023-02-16

**Authors:** Deniz Bedir, Fatih Agduman, Fatih Bedir, Suleyman Erim Erhan

**Affiliations:** ^1^Faculty of Sport Sciences, Erzurum Technical University, Erzurum, Türkiye; ^2^Faculty of Sport Sciences, Ataturk University, Erzurum, Türkiye; ^3^College of Physical Education and Sports, Tekirdag Namik Kemal University, Tekirdag, Türkiye

**Keywords:** curling, performance, team cohesion, empathy, communication skill

## Abstract

Understanding the psycho-social factors such as communication, empathy, cohesion, etc., that affect successful athletic performance is a high priority and primary focus for applied sports psychology. Detailed examination of the athletes’ psycho-social characteristics is essential in revealing which processes play an active role in achieving optimum performance. Developing these features of the athlete can contribute to coordinating the team, sharing tasks, increasing motivation, preparing team members for a change, and improving performance. For this purpose, the mediating role of communication skills in the relationship between empathy, team cohesion, and competition performance was examined in a sample of 241 curlers competing in 69 teams in the Turkish Curling League in the 2021–2022 season. During the data collection process, Personal Information Form, Empathic Tendency Scale, Scale for Effective Communication in Team Sport, and Group Environment Questionnaire were used. Competition performance was calculated by giving 1 point for each match won by the teams in the competitions in which the single-circuit round-robin system is applied. Structural equation modeling was used in data analysis to determine the direct and indirect predictive effects between variables. The study showed that empathy and team cohesion predict competition performance through communication skills, and communication skills fully mediate this relationship. Based on the research results, it was evaluated that communication skills have a substantial effect on the competitive performance of athletes, and this finding was discussed in the context of the literature.

## 1. Introduction

In parallel with the development of sports sciences, the expectation of the highest level of performance from competitive athletes is increasing (Tokdemir, 2011). Athletic performance is the execution of certain physical routines or procedures by someone trained or skilled in physical activity influenced by a combination of physiological, psychological, and socio-cultural factors (Fukuda, 2019). The level of the importance of these factors may differ according to sports branches (Bayraktar and Kurtoğlu, 2004). Since the competition’s success in individual sports depends only on the athlete’s performance, physical and physiological parameters seem to be the key to success. Psychological processes such as team cohesion, communication skills, and empathy are more important in team sports (Kajbafnezhad et al., 2011; Mujika et al., 2018).

The role of psychological skills in increasing performance in sports branches where social, cognitive, and psychological factors are important is one of the most exciting topics for researchers (Birrer and Morgan, 2010; Siekańska et al., 2021). Research in sports settings has provided evidence that psychological skills facilitate athletic performance for both team and individual athletes (Thelwell et al., 2006; Birrer and Morgan, 2010; Beauchamp et al., 2012; Birrer et al., 2012; Hardy et al., 2018). Studies show that communication skills, team cohesion, and empathy are psychological parameters that positively affect competition performance (Sevdalis and Raab, 2014; Cranmer et al., 2020).

The empathy is defined as the process of putting oneself in the place of the other person and looking at events from his perspective, feeling and understanding the feelings and thoughts of the other person correctly, and conveying this situation to him (Dökmen, 2004). Empathy is the basic ability that enables people to notice emotions, establish and maintain relationships with others (Decety and Jackson, 2004). Lack of empathy may be related not only to difficulties in understanding other people’s emotions, but also to difficulties in understanding one’s own emotions (Schipper and Petermann, 2013). This condition is associated with mood disorders, aggressive behavior, and communication disorders in humans (Lovett and Sheffield, 2007; Schipper and Petermann, 2013; Gandhi et al., 2021). The idea that empathy can affect an athlete’s performance has only recently been discussed in the literature. Observing others during physical activity is almost inevitable and exists in settings from recreational physical activity to professional sports, which can affect individual or team performance (Behm and Carter, 2021). The action of observing others fatiguing or in pain due to exercise has been shown in recent studies to elicit a perceptual empathic response (Noakes, 2012; Xu et al., 2019; Yuksel et al., 2019; Astokorki et al., 2021). A more empathetic athlete may contribute to greater team cohesion and spirit, which could be a deciding factor for a coach when choosing between two similarly talented athletes (Behm and Carter, 2021). Remarkably, the athletes competing in team sports should have high levels of empathy to predict their teammates’ reactions, understand the opponent’s emotions, and make the right decision in a dynamically changing environment (Budnik-Przybylska et al., 2021). For this reason, empathy is one of the psychological keys to success in sports environments (Erdem, 2013; Sevdalis and Raab, 2014).

Another psychological factor affecting the competition performance of team athletes is team cohesion. Cohesion has been defined as a dynamic process reflected in a group’s tendency to stay and stay together in pursuit of instrumental goals and/or the satisfaction of members’ emotional needs (Worley et al., 2020). Cohesion, which is defined as an imaginary chain that binds team members together, is a dynamic process that reflects the tendency of a group to stick together and stay together within the framework of common goals (State-Davey, 2009). When we look at the characteristics of successful teams, it is seen that team members are socially close to each other, and their communication is strong (Durdubaş, 2013). The importance of team cohesion becomes more apparent in tasks requiring more group members’ interaction, mutual monitoring, and dependence on each other (Öcal and Aydin, 2009). Many studies on cohesion have shown a positive relationship between team cohesion and team performance (Høigaard et al., 2006; Bell et al., 2019).

Effectiveness communication skills are among the most important ingredients contributing to performance enhancement and the personal growth of sport and exercise participants (Weinberg and Gould, 2019). In addition to affecting performance (Lausic et al., 2009), it is also related to other psychological factors, such as empathy and group cohesion, impacting performance (Carron et al., 2005). In empathy, communication skills are essential in transferring emotions to the other party (D'souza et al., 2020). In this context, interventions, including communication skills training, are applied to develop empathy in many different areas (Stepien and Baernstein, 2006). Effective communication is also associated with team cohesion. Effective communication within the team contributes to the sharing of similar personal stories by the athletes, thereby creating increased awareness and a sense of togetherness (Carron et al., 2002). This situation increases team cohesion by enabling the athletes to cling to each other more (Beauchamp and Eys, 2014). Due to its features, effective communication can mediate the effect of group cohesion and empathy on competitive performance in team sports (Rapisarda, 2002).

Curling is one of the most appropriate branches that can reveal the mediating role of effective communication in the effect of group cohesion and empathy on competition performance because all competitions are played in a single arena and at the same time (Federation, 2020) so that athletes must thoroughly and quickly perceive what their teammates say (the skip’s command to sweep, which shot should be played, and what tactics they apply etc.). For example, when watching a curling match, it can be seen that skips communicate by developing different codes (Woooov: No-sweep, Yeap: Sweep) when they need to give sweep or no-sweep commands to their teammates. During the competition, curlers can communicate with gestures, facial expressions, and verbal communication (Alaeddinoğlu et al., 2022). For example, skips can indicate rock speed with various moves to their teammates. When skip wants a guard shot, he takes the broom between his hands and raises it above his head, and when he wants a take-out shot, he puts his hand on his belly, shoulder, or head. Team members who can better communicate verbally and physically during the game provide more information quickly. Teams that can communicate well are fine with using time in the game (Federation, 2020).

The sport of curling contains more cognitive and psychological skills due to its structure. Curling is the only sport where the route of the stone thrown toward the target can be changed (Buckingham et al., 2006). After the delivery, it is a team sport consisting of athletes who aim to place the stones as close to the target as possible. It is seen that successful teams in the sport of curling consist of players who have played together for many years (Weeks, 2019). Since curling team members consisting of five athletes must train together constantly to ensure consistency in their shots, the athletes must establish close relationships with a limited number of people (Collins and Durand-Bush, 2010, 2016). As a result, athletes competing in curling and other team sports should develop a tactical strategy, make correct and fast decisions, support each other, cope with stress and act in sync in competition as one body. Examining variables such as empathy, communication skills, and team cohesion, which have been scientifically proven to affect individual performance, within the framework of a mediated model in the curling branch will guide researchers and coaches working in this field. In this context, this study aims to examine the mediating role of communication skills in the effect of curlers’ empathy and team cohesion on competition success. In this context, this study aims to examine the mediating role of communication skills in the effect of curlers’ empathy and team cohesion on competition success.

## 2. Methods

### 2.1. Research model

This study, designed in a correlational survey model, examined the relationships between competition performance, empathy, team cohesion, and communication skills in curlers. In this context, Structural Equation Modeling (SEM) was used to explain the predictive correlations between variables (Collier, 2020).

### 2.2. Participants

A total of 241 athletes participated 112 males (46.6%) and 129 females (53.5%) from 69 teams competing in the Turkish Curling League in the 2021–2022 season in the study. Ages of the athletes ranged from 18 to 38 (x- =26.08 ± 6.81), between 1 and 11 years of licensed sports (x- =5.86 ± 2.82), and between 1 and 10 years of playing with teammates (x- =3.48 ± 2.00). Other descriptive information about the athletes participating in the research is given in Table 1.

**Table 1:** Descriptive of the participants.

Variable	*n*	%
**Gender**		
Male	112	46.5
Female	129	53.5
**Order**		
Lead	56	23.2
Second	42	17.4
Third	79	32.8
Fourth	64	26.6
**Position**		
Skip	84	34.9
Vice	73	26.1
Front – end	94	39.0

Since the collected data includes information about the team, such as intra-team communication and team cohesion, the participation of at least two athletes from each team was determined as the criterion for inclusion in the research.

### 2.3. Data collection tools

The data collection tools in the research consist of 5 parts: Personal Information Form, Empathic Tendency, Team Cohesion, Effective Communication Scale for Sports Teams, and Turkey Curling League competition (performance) scores for the 2021–2022 season.

#### 2.3.1. Personal information form

In the personal information form prepared by the researcher, some items include the participants’ gender, age, order, and position information in the team.

#### 2.3.2. Empathic tendency scale

The Empathic Tendency Scale developed by Dökmen (1988) was used to determine the empathy potential of the athletes participating in the study and the level of empathic behavior in daily life. The scale consists of 20 items prepared as a Likert-type scale. In the scale, there are questions to measure the empathic tendencies of the individual, such as “The problems of others concern me as if they were my problems,” “My close friends often tell me about their problems,” “When arguing with someone, sometimes my attention is focused on my answers rather than what they say.” The scores of the scale items are “strongly agree = 5” and “strongly disagree = 1” and the possible scores are between 20 and 100. A high score means a high empathic tendency, and a low score means a low empathic tendency. The Empathic Tendency Scale was administered by Dökmen (1988) to a group of 70 students twice, 3 weeks apart. The reliability coefficient of the test repetition obtained from these two applications was found to be 0.82. The correlation coefficient between the scores the students got from the odd and even items of the scale with the split-half method is 0.81. For the validity study, the relationship between the Empathic Tendency Scale and the “Understanding Emotions” section of the Edwards Personality Preference Schedule was examined. A correlation of 0.68 was found (Çevik, 2017). In this study, the Cronbach Alpha reliability coefficient of the scale was found to be 0.934.

#### 2.3.3. Scale for effective communication in team sport-SECTS

Scale for Effective Communication in Team Sport (SECTS), developed by Sullivan and Feltz (2003) and adapted into Turkish by Alkan (2009), was used in the study to determine the communication skill levels of athletes. SECTS is a 7-point Likert-type scale comprising 15 items measuring effective communication skills within a team. It has four sub-dimensions: Acceptance, Distinctiveness, Positive Conflict, and Negative Conflict. Acceptance is measured by three items and refers to interpersonal messages of support or evaluation (for example, we trust each other). Distinctiveness is measured by three items and includes these messages of a shared, overarching team identity (for example, we use aliases). Positive Conflict refers to a constructive, emotionally controlled discussion of interpersonal differences (for example, compromising with one another when we disagree). In contrast, Negative Conflict refers to disagreements expressed in a confrontational or destructive way (for example, we look each other in the face when we disagree). While these four dimensions vary widely within sports teams, they are related to team cohesion (Sullivan and Short, 2011) and performance (Ji and Yan, 2020). Confirmatory Factor Analysis (CFA) validated the scale with a sample of 251 athletes. The CFA showed that the model fit this sample strongly as well: x2/df = 1.97, CFI = 0.94, GFI = 0.91, GFI = 0.91, AGFl = 0.87, RMR = 0.03, and RMEA = 0.06. Goodness of fit indicates an excellent model, especially CFI, RMR, and RMSEA (Browne and Cudeck, 1992; Shi et al., 2019) and chi-square ratio (Mertler et al., 2021). The Cronbach Alpha values of the sub-dimensions of the original scale were Negative Conflict: 0.69, Acceptance: 0.86, Distinctiveness: 0.84, and Positive Conflict: 0.73.

#### 2.3.4. Group environment questionnaire-GEQ

The “Group Environment Questionnaire” developed by Carron et al. (1985) and adapted into Turkish by Öcel and Aydin (2006), was used to determine the team cohesion levels of the participants. This 18-item instrument quantifies task and social cohesion, maintaining a distinction between individual-attraction-to-group, and group-integration cohesion, distributed among four subscales. ATG-task cohesion quantifies how firmly each player is drawn to the group to satisfy individual task completion needs. ATG-social cohesion measures how strongly each player is attracted to the group to meet personal, social, and friendship needs. GI-task cohesion assesses the extent to which team members unite successfully to complete the common performance goal. GI-social cohesion quantifies perceptions of the degree to which the team bonds to satisfy social desires. The GEQ has been utilized extensively in the sports psychology literature as the instrument’s psychometric properties are well established (Carron et al., 1985, 2005). Internal reliability for the ATG-task, ATG-social, GI-task, and GI-social cohesion subscales was 0.81, 0.75, 0.73, and 0.83, respectively (Carron and Ramsay, 1994).

### 2.4. Performance evaluation

Thirty-five male and 34 female teams participated in the 2021–2022 Turkey Curling League competitions according to the single round-robin system, and the teams received 1 point for each match they won, just like in the curling league. No points were awarded to teams for lost matches.

### 2.5. Procedure

The researchers met with all the athletes participating in the research (with each team separately) at Erzurum Curling Hall the day before the championship (on the last training day). After the data collection tools were introduced, the participants filled out the self-report scales. The performance scores used in the research were obtained from the Turkish Curling Federation web page. Before analyzing the research data, the researchers subjected it to a preliminary analysis to check whether it be missing or incorrect data. As a result, seven athletes, four females and three males, who filled out the form incorrectly, were excluded from the evaluation. In addition, two clubs that filled the data collection tools with a single athlete were excluded from the study.

### 2.6. Data analyses

Structural Equation Modeling (SEM) was used to test the research hypothesis. Structural equation modeling is a set of techniques in which latent structures can be examined through observable variables (Jöreskog and Sörbom, 1996). SEM was ideal for the correlational analysis targeted by the research, as it not only reveals the parameters of the correlation between latent variables but also allows the determination of error variances. The data obtained from the scales were transferred to electronic media using the SPSS package program. Descriptive statistics and correlation analysis of the variables were done with the SPSS 24.0 program, and the model test was done with the AMOS 24.0 program. “Maximum Likelihood (ML)” and “Covariance Matrix” were used as parameter estimation methods.

The analysis phase examined whether the data obtained primarily met the normality assumptions necessary for establishing the structural equation model. For this purpose, extreme values were examined using the z-test, kurtosis, and skewness coefficients. Mahalanobis distance coefficients were examined for the multi-directional outliers and the one-way outlier analysis. After analyzing the one-way and multi-directional extreme values, the normal distribution assumption was examined with the Kolmogorov–Smirnov normality test. The data set showed normal distribution characteristics. After testing the normal distribution assumptions, the variance inflation factor and auto-correlation were examined before the analysis. It was observed that there was no auto-correlation and that the variance inflation factors were within the required limit values. Afterward, it was decided that the data set was suitable for parametric statistical analysis, and the data analysis was started.

Mediated structural model testing consists of three main stages (Baron and Kenny, 1986). These steps are as follows; (i) Exogenous variables (empathy and team cohesion) should significantly predict endogenous variables (competition performance). (ii) The effect of exogenous variables on the mediating variable (communication skills) should be significant. (iii) The relationship between the mediator and endogenous variables should be significant. After all the criteria are met, when the mediator variable is included in the model that explains the relationship between endogenous and exogenous variables, there is expected to be a significant decrease in the amount of the relationship in the first stage, or it will lose its meaning. The disappearance of the relationship between endogenous and exogenous variables indicates that the mediating variable is strong and unique (full mediation effect). Although there is a significant decrease in the level of the relationship, the relationship still retains its meaning, indicating a partial mediation effect.

## 3. Results

During the analysis of the data, first of all, correlation analysis was performed in order to test the relationships between the variables in the research. Relationships between variables are presented in Table 2.

**Table 2:** Investigation of the relationship between variables by Pearson product–moment correlation.

	*n*	*M*	*SD*	1	2	3	4
1. Performance point	241	10.78	5.40	-			
2. Empathy	241	4.30	1.06	0.600**	-		
3. Cohesion	241	4.96	1.18	0.761**	0.716**	-	
4. Communication skills	241	3.71	0.71	0.707**	0.615**	0.702**	-

**p < 0.01.

In Table 2, it was found that there was a statistically significant relationship between all the variables in the study.

After examining the relationships between the endogenous and exogenous variables of the study, a two-stage method was followed for the mediation analysis with the structural equation model (Anderson and Gerbing, 1988). In the first stage, a measurement model was created with confirmatory factor analysis, and thus, it was tested whether the relationships between latent and observed variables were acceptable or not. Then, the relationships between latent variables were tested with the structural model.

Validation measurement models are essential for testing structural equation models (Şimşek, 2020). The fit indices obtained from the measurement model are as follows: [*χ*^2^/sd = 1.85; CFI = 0.92; GFI = 0.91; SRMR = 0.09; RMSEA = 0.06]. These show that all latent variables fit well with the indicator variables and other latent variables they represent (Tabachnick et al., 2007).

**Figure 1:**
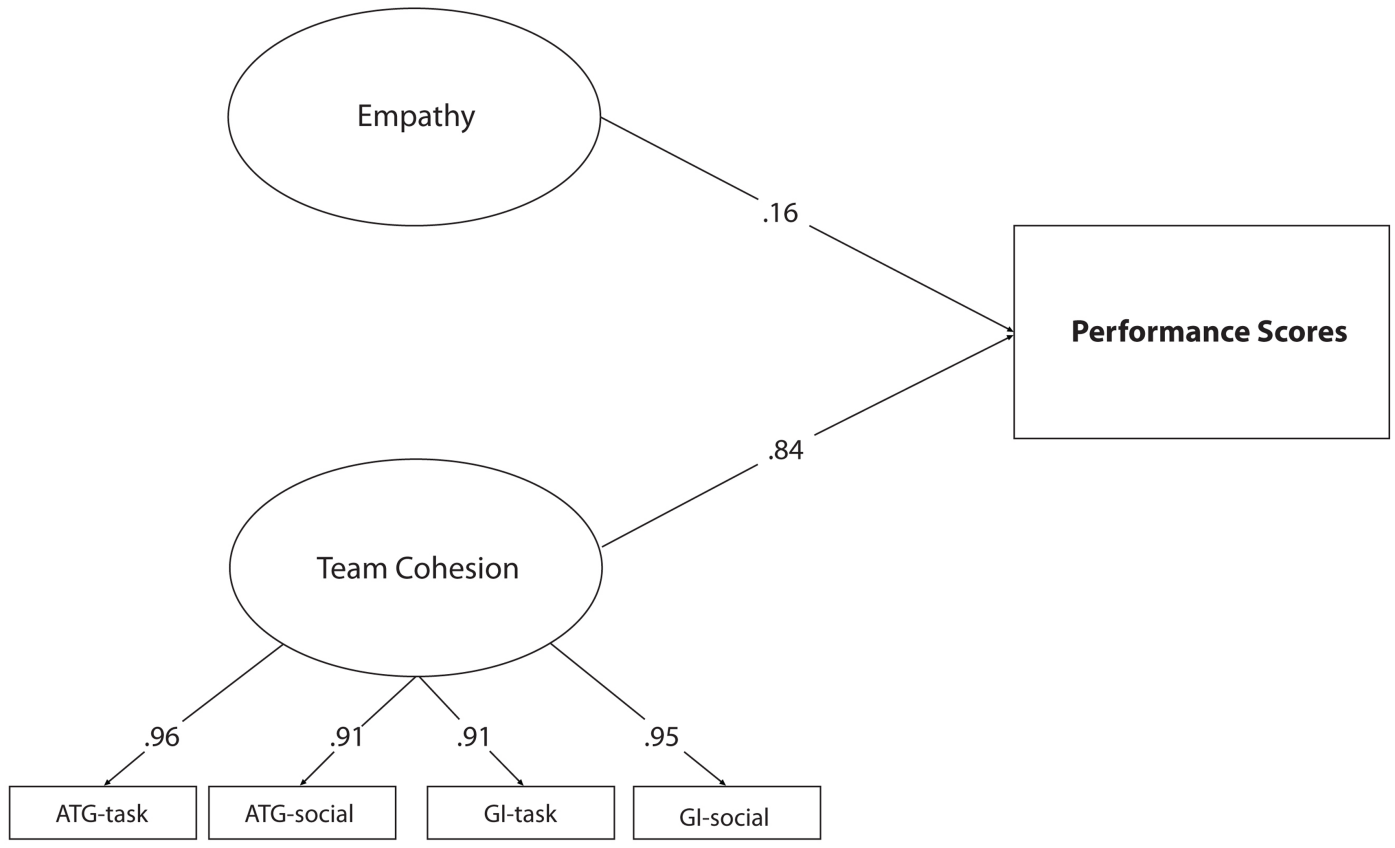
Standardized SEM results for Model 1.

After verifying the measurement model, three different models created for the research were tested, respectively. In this context, Model 1 tested the direct predictive effects of empathy and communication skills on athletes’ competitive performance. Findings related to Model 1 are presented in Figure 1.

Considering the model whose fit indices [*χ*^2^/sd = 1.81; CFI = 0.94; GFI = 0.91; RMSEA = 0.06, SRMR = 0.060] were tested in Figure 1, it can be said that all latent variables in Model 1 have a significant relationship with the observed variables they represent (*p* < 0.01). Model 1 shows that empathy and communication skills positively predict competition performance (*β* = 0.15, *p* < 0.01, *β* = 0.84, *p* < 0.01).

**Figure 2:**
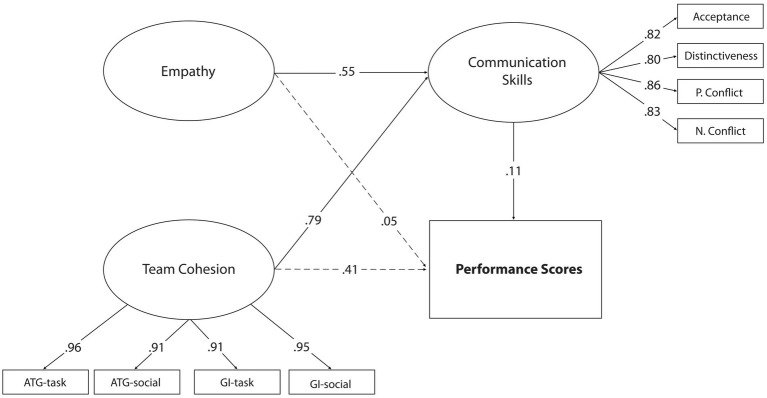
Standardized SEM results for Model 2.

After confirming the hypothesis in Model 1, the second stage of mediation relations should be applied. At this stage, the mediation effect of the model is included, and the parameters related to the direct and indirect relationship processes between the predictor variables and the predicted variable are examined. In this context, the model designed in Model 1 included communication skills between empathy, team cohesion, and competition performance and was tested as Model 2. Findings for Model 2 are presented in Figure 2.

**Figure 3:**
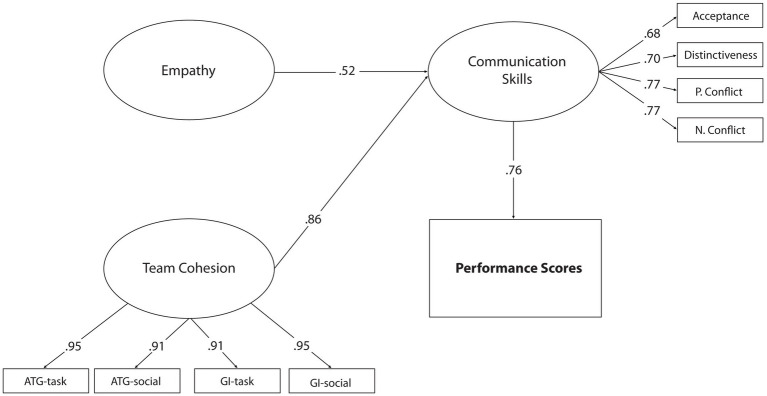
Standardized SEM results for Model 3.

According to the model in Figure 2, *χ*^2^/df = 1.64 was significant at the *p* < 0.01 level. It was determined that the goodness of fit indices of the structural model (RMSEA = 0.076, CFI = 0.92, GFI = 0.94, SRMR = 0.065) were at an acceptable level (Tabachnick et al., 2007; Çokluk et al., 2012). These values show that the established structural model is a good fit. Thus, the hypothesis that communication skills mediate the relationship between empathy and team cohesion and competition performances have been confirmed. According to this result, empathy, team cohesion, and communication skills explain 53% of the total competition performance. On the other hand, when Figure 2 is examined, empathy had a significant effect on competitive performance (*β* = 0.16 *p* < 0.01) in Model 1, but this significant relationship disappeared after the team cohesion variable was included (*β* = 0.05, *p* > 0.05). Similarly, in Model 1, while team cohesion had a significant effect on competition performance (*β* = 0.84, *p* < 0.01), its effect decreased after the communication skills variable was included in the model (*β* = 0.41, *p* < 0.05). With the addition of the team cohesiveness variable to the model, a significant change was observed in the correlation coefficients between the variables, which can be considered a strong indication that there may be mediation relationships. In addition, when Figure 2 is examined, it is seen that the predictive effect of communication skills on competition performance is insignificant if there are direct and indirect ways between the variables (*β* = 0.11, *p* > 0.05). Therefore, the full mediation role of the communication skills variable was tested by subtracting the direct paths to competitive performance from the empathy and team cohesion variables. This model, called Model 3, deals with full mediation relationships between variables. The structural model that considers the full mediation relationship is presented in Figure 3.

Model 3 indices and parameters [*χ*^2^/df = 1.65; RMSEA = 0.055; CFI = 0.95; GFI = 0.93; SRMR = 0.052] show that the pattern under test and communication skills mediation is confirmed. When Figure 3 is examined, it is seen that empathy (*β* = 0.52, *p* < 0.01) and team cohesion (*β* = 0.86, *p* < 0.01) predict competitive performance through communication skills. Considering the model 2 parameters, it is understood that there are improvements in statistical values after the paths showing low or insignificant predictions are removed from the model. For this reason, the good fit of the hypothesized model and the observation of a significant change in the path coefficients between the variables were accepted as indicators of the mediating role of communication skills. In addition, the predictive effect of communication skills on competitive performance was determined as *β* = 0.76, *p* < 0.01. Compared to Model 2, it is thought that there is a significant increase in the predictive coefficient of team cohesion (*β* = 0.11, *p* < 0.01) on competitive performance. These values are obtained by removing low or insignificant relationship paths in Model 2 from the model.

## 4. Discussion

Increasing competition in the sports world has caused athletes to approach each other regarding physical and physiological characteristics (Ostojic et al., 2006; Sporis et al., 2009; Torres-Luque et al., 2016). The importance of psychological factors has increased in the ability of athletes with similar technical, tactical, and power parameters to be superior to each other (Weinberg and Gould, 2019). Revealing the relationship between psychological parameters that affect performance will shed light on athletes and coaches. In this context, the research aimed to determine the mediating role of communication skills in the effect of empathy and group cohesion on competition performance.

The effect of predictive variables on the competitive performance of curlers can be discussed in two ways, according to the research findings. The first category is direct effects, and the second category is indirect effects. The direct effects of empathy and group cohesion on competitive performance can be discussed. As indirect effects, the predictive effect of empathy and group cohesion on competitive performance can be discussed *via* the communication skills variable.

The results of the study showed that empathy has a positive effect on competitive performance. Neurologically, empathy is explained by the common coding principle, which assumes a bidirectional link between perception and action (Gentsch et al., 2016). This aspect of empathy allows the observer to put himself or herself in the other person’s shoes with associated neural, autonomic, and somatic responses to events experienced by another person (Preston and De Waal, 2002; Biven and Panksepp, 2007; Preston et al., 2013). This approach is consistent with the principle of standard coding, which assumes a bidirectional link between perception and action in terms of shared perceptual-motor representations and shared resources in the functional architecture of the brain (Hommel et al., 2001; Decety and Meltzoff, 2011). Empathy may be an essential psychological skill for success in sports competitions, as athlete performance requires a bidirectional link between perception and action and a high level of expertise and shared resources in the brain’s functional architecture (Sevdalis and Raab, 2014). For example, in team sports, athletes must replace an opponent or teammate to anticipate their reactions, understand the opponent’s and teammates’ emotions, imagine their reactions, and make the right decision in an instantaneously changing environment (Budnik-Przybylska et al., 2021). The athletes’ morale can deteriorate in curling, especially after unsuccessful shots. In such cases, athletes with high empathic tendencies can help increase performance by preventing their teammates from psychologically dropping out of the game (Shima et al., 2021).

They can be considered aspects of a close link between perception and action, in the sense that a curler’s understanding of the cognitive and emotional aspects of the target (stone, teammate, opponent) may be related to the degree to which he empathizes with that target while experiencing actions, emotions, and somatosensations (Keysers, 2011; Sevdalis and Raab, 2014). Jola et al. (2012) used transcranial magnetic stimulation (TMS) to measure corticospinal excitability *via* motor-evoked potentials (MEPs) in both hands and arms while watching participants’ dance moves. The results showed that for watchers who had visual experience in a particular dance style (i.e., Indian dance), higher scores in fantasy were positively associated with more excellent MEPs in muscle-controlling arm movements. This result completes the relationship between activity in the action-observation network and aspects of empathy. Empathic tendencies may be associated with positive attitudes and emotions in interpersonal settings. In coach-athlete dyads, positive perceptions of a partner’s viewpoint were associated with empathic accuracy for both coaches and athletes; ultimately, increased empathic accuracy was associated with higher levels of satisfaction with training, though only for athletes (Lorimer and Jowett, 2009).

This potential can be exploited using designs that evaluate empathy in different performance areas. For example, empathy in sports environments; (i) performance can be used to optimize empathy (by providing appropriate educational environments, experiences, and interventions) (ii) or vice versa to optimize performance (for example, by improving behaviors, interactions, and relationships to promote desired positive effects) (Sevdalis and Raab, 2014). In addition, although empathy had a relatively low predictive effect on competitive performance in Model 1, this effect increased in Model 2, where communication skills were included in the analysis. That is, empathy strongly influences competition performance through the communication skills variable.

When the communication skills variable is included in Model 2, the disappearance of the direct predictive effect of empathy on competition performance shows that communication skills have a mediating role, and type II error is prevented. Empathy is an essential force behind many positive prosocial and social behaviors that foster team cohesion and cooperation (Johnson et al., 2005; Jolliffe and Farrington, 2006; Luo and Xie, 2018). Lack of empathy can play a critical role in developing group members’ exclusion and other behavioral problems in team sports (LeSure-Lester, 2000; Jolliffe and Farrington, 2006). Since the coach cannot directly interfere with the game, the athletes take various initiatives in curling. As a result, they sometimes fail and want to see their teammates support them in every situation. Because only in this way can they make a successful shot in the next attempt. In team sports such as curling, where the level of empathy and team cohesion is low, the psychological pressure on the athletes can cause them to fail (Shima et al., 2021).

The second variable that directly and indirectly affects competition performance is team cohesion. The study showed that team cohesion positively and strongly predicted competition performance in curlers in Model 1. However, there was no significant change in the prediction coefficient with the inclusion of communication skills in Model 2. The results suggest that team cohesion predicts competition performance directly and indirectly through communication skills. Therefore, the continuation of the direct effect after the inclusion of communication skills in the model indicates a partial mediation relationship. This result is in line with the research that shows that team cohesion positively affects performance (Braun et al., 2020; Grossman et al., 2022). There is evidence in the literature that highly cohesive teams show a significant relationship with performance, as interactive sports require a high level of task dependence (Mach et al., 2010; Eys and Kim, 2017; McEwan, 2020). A meta-analysis of team environments found that the relationship between cohesion and performance in sports teams was significantly more vital than in other work teams (Carron et al., 2002). Sports teams are an example of close-knit groups that must work together to succeed. Team commitment improves coordination, effort, and results (Cranmer et al., 2020).

Kim et al. (2016) investigated the relationship between communication and group cohesion. They revealed that intra-team communication has a significant relationship with group integration-task and group integration-social sub-dimensions of team cohesion. It is claimed that intra-team communication creates conditions for higher levels of emotional commitment among team members, thus increasing team cohesion (Lauring and Selmer, 2010; Smith et al., 2013). So compliant teams tend to work more effectively and perform better than less compliant teams (Barrick et al., 2007; Smith et al., 2013).

The findings show that communication skills mediate the relationship between empathy, team cohesion, and performance. Effective intra-team communication can assist the athletes of an interactive sports team by directing (i.e., planning), encouraging (i.e., motivating), and evaluating (i.e., appraising) the performance of each member (Cunningham and Eys, 2007). In team situations where conflict is likely, recognizing and resolving conflict is crucial to team success. Choi et al. (2019), who examined how effective communication skill affects the climate within the team, revealed that although there is a positive correlation between effective communication and coach-athlete relationship and team effectiveness, there is a negative relationship with aggression. Ineffective communication can cause individuals to dislike, lose their trust, refuse to listen to each other, cause differences of opinion, and cause many more interpersonal problems within the team (Whetten and Cameron, 2016). A US-based study listed linguistic features such as coach communication, frequency of pauses, repetitions, verbs, and jargon during training for junior women’s basketball teams (Masterson et al., 2006). The most important among these studies is the analysis of the relationship between the points received in the doubles tennis match and the communication established (Lausic et al., 2009). Couples’ speech between play sets was coded as ‘emotional’ or ‘action’ expressions. Winning couples were found to exchange twice as many messages as losing couples, and overall communication patterns were more homogeneous than those losing couples typically exhibit. The same is true for the sport of curling. Successful team athletes in the sport of curling talk about what kind of strategy will be applied at the next end during the 1-min break between the ends and evaluate the plans to be implemented in the end. The results obtained support the model created in the research.

In sports such as curling, strategy and motivation are highly dependent on maintaining on-court communication (Alaeddinoğlu et al., 2022). Athletes who undertake the sweeping task in curling instantly inform the skip (by shouting) about where the stone will stand on the playing field after the stone is thrown. Thus, the skip gives feedback on whether the in front of the stone should be swept. Therefore, it can be concluded that the athletes of the teams that can communicate more effectively during sweeping will be more accurate. Sports events are intense and stressful environments where athletes with different goals, abilities, social structures, and communication skills come together. It is known that effective communication in the sports environment increases the athletes’ effectiveness, helps them establish relationships with other athletes, and improves their motivation positively (Choi et al., 2019).

## 5. Theoretical contribution

It is expected that the communication skills of successful individuals in the field of art and sports will be highly effective, as is the case in occupational groups such as health, education, and management, which are in constant communication with different people. The results obtained are essential in showing that the athlete’s good communication with his teammates and environment is a process reflected in the athletic performance.

Evidence has been strengthened that the relationship between communication skills and predicting what the opponent and teammates think in the competition makes the athlete more advantageous. Therefore, athletes with high empathy levels are closer to success.

Since the psychological aspects of athletic success involve very complex processes, there may be a better approach to reveal the factors affecting success because different psychological processes affect each psychological skill. Although empathy and team cohesion have a positive effect on athletic success, this effect is further increased by the intermediary role of communication skills. These results have the feature that can contribute to revealing the complex psychology-performance relationship.

On the other hand, competition performance can also be considered in terms of sport type. In individual sports, it is much easier to evaluate performance, determine the factors affecting it, follow its development, and make performance-improving suggestions compared to team sports (Özkara, 2002). For a group of athletes to be called a team, interaction, information exchange, and an emotional bond must be formed for the same purpose. It is expected that the higher the density of these elements, the higher the group dynamic will be (Aydın, 2018). The results show that for high performance in team sports, team members must have a high level of harmony, empathy, team cohesion, and communication skills.

## 6. Limitations and future recommendations

The most important limitation of the study is that only the teams competing in the Turkish Curling League are included in the research, and international athletes are not included in the research. In future studies, a wider group of participants can be formed by including curlers with similar expertise from different countries. In addition, only curling sport was included in the study. The results obtained by applying the model created in the research to different team sports can be compared.

In order to reveal the psychological parameters that affect the competition performance, a new and more comprehensive model can be created with different emotional intelligence elements apart from empathy and communication skills.

## Data availability statement

The datasets presented in this study can be found in online repositories. The names of the repository/repositories and accession number(s) can be found below: https://doi.org/10.48623/aperta.252080.

## Ethics statement

The studies involving human participants were reviewed and approved by This research was designed in accordance with the Helsinki Declaration and was approved by the Atatürk University Faculty of Sports Ethics Committee (no: 70400699-000-E.2100023787, date: 25.01.2021). The patients/participants provided their written informed consent to participate in this study.

## Author contributions

DB organized the study. DB, and FA collected the data. DB, FA, FB, and SE designed the methods and wrote the first draft of the manuscript. All authors contributed to the article and approved the submitted version.

## Conflict of interest

The authors declare that the research was conducted in the absence of any commercial or financial relationships that could be construed as a potential conflict of interest.

## Publisher’s note

All claims expressed in this article are solely those of the authors and do not necessarily represent those of their affiliated organizations, or those of the publisher, the editors and the reviewers. Any product that may be evaluated in this article, or claim that may be made by its manufacturer, is not guaranteed or endorsed by the publisher.
